# Music motivation depends on what to motivate: research review of Gumm’s music teaching and conducting models

**DOI:** 10.3389/fpsyg.2023.1293872

**Published:** 2023-11-23

**Authors:** Alan J. Gumm

**Affiliations:** School of Music, Central Michigan University, Mount Pleasant, MI, United States

**Keywords:** music teaching, music conducting, music motivation, music performance, comprehensive musicianship, music education philosophy

## Abstract

The history and philosophy of music education are traced with varied efforts to hone, enhance, and shift a strong tradition of performance-based instruction. The purpose of this study is to summarize the research of Gumm’s empirical models of eight music teaching and six conducting approaches and their application in the profession across three decades toward varied philosophical aims. Each approach coordinates a distinct set of instructional and motivational behaviors toward a particular learning effect or outcome. Different balances of approaches reveal broader aims, such as performance, comprehensive musicianship, cooperative, discovery, and affective learning, or even basic on-task behavior. Broader yet are two overarching aims found in common to both music teaching and conducting—to control or release. Controlling music teaching asserts correct on-task behavior through clear task directions and corrective feedback, motivates attention to task nonverbally, efficiently fills time with active tasks, and clarifies and affirms positive learning. In contrast, cooperative group leadership releases interdependent learning, questioning fosters music concept learning, imagery and movement release artistry, and discussing unique perspectives releases independent ideas and feelings. In conducting, precise gestures control accurate timing, signals and alerts motivate attention, and mimicry of musician exertions controls physical tone production; whereas shaping of phrases and score markings release musical expression, psychosocially familiarized gestures foster interdependence, and tension-easing gestures release freer independent tone production. Control-oriented teaching is most prevalent across the field, yet links to greater burnout and appeals to accommodating students motivated by effort and ability more than students motivated by social and affective enjoyment of music. In conducting, music-oriented precision and expression are more prevalent than musician-oriented approaches. Releasing approaches are more prevalent in Western than Eastern culture, upper levels, early rehearsals well before concerts, smaller ensembles, competitive ensembles, and teachers and conductors with greater experience or varied movement training. Conclusive implications are that the key to motivation is to draw attention to specific and intentional forms of learning, and that whatever is motivated to attention also motivates a particular philosophy of music education. Future research is suggested in general music and ethnic, folk, popular, community, and professional music ensembles.

## 1 Introduction

The history and philosophy of music education are traced with varied efforts to hone, enhance, and shift a strong tradition of performance-based instruction. Starting in the 1970s, music education research sought to sort out how to effectively keep behavior focused on directed performance tasks and meet comprehensive and alternative aims (Gumm, 1992). Most theories focused on a single set of dichotomous aims, such as unsupportive vs. supportive (c.f., direct vs. indirect, formal vs. informal, or teacher- vs. student-oriented), high vs. low magnitude or intensity, complete vs. incomplete sequential patterns (i.e., task directions, student response, reinforcing feedback), and most to the point, music performance vs. comprehensive musicianship. Also, starting in the 1970s, research on conducting sorted out music-related gestures as well as nonverbal kinesics of conducting that extended to everyday gestures relatable to sign language and mime (Gumm et al., 2011). Music conducting theories focused chiefly on dichotomies of expressive vs. mechanical or inexpressive gestures, high vs. low intensity as an overlapping concern, and gestures that induce vs. ease tension in physical technique.

In Gumm (1993) and Gumm et al. (2011), I initiated a more holistic multivariate approach that led to models that remain the most comprehensive in the field. The music teaching model, comprised of eight factors, is encapsulated in the *Music Teaching Style Inventory* (MTSI; Gumm, 2009). The conducting model comprises six factors, is encapsulated in the *Conducting Priorities Survey* (CPS; Gumm, 2016c), and is affirmed as having been developed through the “most systematic research on the functions that the conductor fulfills” (Jansson et al., 2022, p. 513).

The purpose of this study is to summarize research on the varied approaches of these models and their application in the profession across three decades toward varied philosophical aims. Compared to practical pedagogical summaries of the music teaching model (Gumm, 1994, 2003a, 2005a,b) and conducting model (Gumm, 2012, 2020), this is a review of all research studies found to have collected data on the two models—my own 13 survey and mixed-methods studies and nine additional researcher applications of surveys that also include mixed-methods comparisons with qualitative observation and interviews.

As empirical models, I did not invent them through personal experience, expert opinion, or subjective sorting. Rather, each approach is a statistical coalescence of prior research content based on the self-reported behaviors of thousands of practicing music teachers and conductors. As a measurement instrument, the MTSI has been tested for content and construct validity in elementary, lower secondary, upper secondary, and college/university general, choral, and instrumental music settings both across the U.S., (Gumm, 1993, 2004a,b, 2007; Gumm and Essmann-Paulsen, 2001; Basilicato, 2010; Groulx, 2010; Olesen, 2010; Bazan, 2011) and internationally (Tsai, 2000; Shah, 2005, 2007; Hsieh, 2010). Also verified in research is its predictive validity with festival and competition ratings (Gumm, 2003b; Groulx, 2010), discriminant validity with measures of learning style, motivation, teacher burnout, and personality (Gumm and Essmann-Paulsen, 2001; Gumm, 2004a; Basilicato, 2010; Groulx, 2010; Gumm and McLain, 2013), and convergent validity against college student evaluations of ensemble director effectiveness, objective video observations, and teacher/director and ensemble musician interviews (Gumm, 2004b, 2007, 2018; Bazan, 2011; Gumm et al., 2018). The CPS similarly has been validated through mixed-methods research and regional and national surveys of conductors of varied ensemble types, educational levels, and career experience, including discriminant validity and relations with MTSI scores (Gumm, 2016a,b, 2018; Gumm et al., 2018).

Certainly, though, in interpreting statistical results, I drew upon prevalent terminology and understandings that, at this point, warrant clarification. As a first clarification, I recognize the inconsistency of developing a model of music teaching style dimensions versus a model of conducting functions, each drawn from respective research trends at the time. For consistency, in this writing, I address both issues the same for both models, referring to dimensions as *factors* or *approaches* and their functions as *aims*, *intentions, focus*, or *motivations*. In discussing the unique aim underlying a particular balance of approaches, I substitute the words *priority*, *balance*, and *practice* in place of style. First, we look at the varied, effective approaches to music teaching and conducting.

## 2 Approaches

### 2.1 Patterns of behavior

I originally posed a definition of music teaching style as patterns of behavior (Gumm, 1993). To bring this up to date, music teaching and conducting approaches moreover are *coordinated* and *stable* patterns of behavior.

#### 2.1.1 Coordinated patterns

The notion of coordinated behavior patterns is similar to the theory of sequential patterns or units of music instruction as complete or incomplete depending on whether each behavior is carried out (Yarbrough and Price, 1981, 1989). This notion is reflected in the research methods I selected to develop both the music teaching and conducting models.

By the factor analysis methods applied, music teachers and conductors participating in theory development had to have distinctively and consistently rated behaviors in coordination for them to coalesce into factors. In theoretical terms, each factor represents latent traits or constructs that are more considerable than defined by measurable behaviors. MTSI and CPS survey items are merely the most reliable and representative measures among a large set of related behaviors, and all coordinated around a distinct global construct.

By survey design, then, high scores result when each behavior or gesture in the pattern is used most frequently, with moderate scores resulting from incomplete or *uncoordinated* use and low scores resulting from low frequency or nonuse overall. Table 1 shows the most representative survey content for each approach.

**Table 1:** Music teaching and conducting factors and most definitive content.

Music teaching model			
Control	Assertive Teaching Give clear task directions. Monitor behavior closely. Communicate an awareness of student behavior. Remind to follow directions. Verbally demand sharp attention to tasks. Give corrective feedback to responses to directions.	Time Efficiency Give directions as quickly as possible. Require students to act quickly to directions. Keep students busy and active. Keep a brisk pace of activities. Get as many things done in scheduled time as possible.	
	Positive Learning Environment Support and care about student feelings. Clarify information that students are uncertain about. Take time to answer student questions. Allow students to answer questions completely. Praise students when they do a good job.	Nonverbal Motivation Change intensity or pace of activity. Change proximity/closeness to musicians. Attention-getting eye contact, facial expressions, body stance, and hand gestures. Show enthusiasm.	
Release	Group Dynamics Have students rehearse/learn in small interactive groups. Have students work with each other. Have individuals present and perform for peers. Have the group be led by student leaders. Have students brainstorm among themselves.	Artistic Music Performance Describe/compare musical events in physical, visual, aural/sound terms. Refine internal music images. Describe music in metaphors. Use physical movement methods.	
	Music Concept Learning Present concepts about music performed. Ask to recall and recognize music terms/facts. Ask to draw comparisons between examples. Ask to diagnose problems in own performance. Ask to solve a musical/music-making problem.	Student Independence Use discussion and dialogue. Develop unique ideas about music. Encourage students to be creative. Ask students to explore how they feel about music.	
Music conducting model			
	Music-oriented	Musician-oriented	
Control	Mechanical Precision Gesture distinct meter patterns. Provide clear downbeats. Indicate clear beat points. Give distinct cues and cutoffs. Indicate precise tempos and tempo changes.	Motivational Maintain eye contact. Shift gaze to alert attention. Circulate to closer proximity. Signal reminders ahead of musical events.	Physical Technique Model healthy technique and stance. Gesture proper skills for musicians to copy. Guide movement size, strength, & energy. Mirror musicians’ physical motions.
Release	Expressive Depict expressive markings in the score. Change dynamics in right or independent left hand. Shape phrase contours and peaks. Gesture the music’s overall expressive character. Reflect the emotional intent of the music.	Psychosocial Use gestures made familiar to musicians. Use gestures that vulnerably respond rather than control. Mime familiar objects or actions portraying the music. Choose gestures based on ensemble member ideas.	Unrestrained Tone Use gestures that ease tension in musicians’ performance. Gesture for musicians to release an upward lifting tone. Shape the flow of ensemble tone. Minimize motion so musicians learn to follow each other’s influences. Stop conducting for musicians to develop their own internal tempo.

#### 2.1.2 Stable patterns

From the start, in both lines of research, I further sought to examine stable, pervasive, consistent patterns of music teaching and conducting behavior. As originally reviewed (Gumm, 1992) and then revisited in critique (Sprikut, 2015), researchers have long been divided into whether teaching style is quick-to-change on a whim or stably slow to change. In initial reviews of research (Gumm, 1992, 1993), I detected a certain preoccupation in music education research with shifting teaching behaviors and single-aim approaches to the neglect of stable overarching patterns and trends. I initiated a research line to fill this void.

Methodological choices reflect an aim to measure stable factors. In both the MTSI and CPS, behaviors and gestures are rated on a scale of never to always to establish how persistently they are used across time. Items related to each approach are then summed—each set representing a distinct pattern of behaviors or gestures. Moreover, latent traits identified through factor analysis are defined as stable ways people behave that may change slowly with experience.

Finally, the ability of the MTSI and CPS to measure stable approaches was validated in research. Test–retest applications of the MTSI validated the stability of approaches after months of teaching (Gumm, 1993). Correlations between university conductors’ CPS self-ratings, content analyses of conductor video-recall interview descriptions and my expert observation analysis of the same series of randomly selected rehearsal episodes (Gumm, 2018) relatively support that the CPS captures stable, consistent approaches. Similarly, Bazan (2011) found how interview self-descriptions of music teaching approaches used across a concert season balanced out similarly to MTSI scores—answering a puzzle of not seeing a balance in limited rehearsal observations. Conclusively, the CPS and MTSI capture stability that is only externally observable across a representative sampling of rehearsals.

### 2.2 Underlying aims

Patterns of behavior not only coordinate into distinct approaches but are coordinated toward particular learning *aims*. The aim behind each behavior pattern I determined interpretively, informed by closest matching prior theories and approaches (Gumm, 1993; Gumm et al., 2011). Table 2 lists the learning aims interpreted by each music teaching and conducting approach.

**Table 2:** Music teaching and conducting approaches, priorities, aims, and influences.

Model Focus^†^		Approach	Learning aim	Background influences	Method influences *
Music teaching	Control	Assertive Teaching	Correct to task	Lower levels, instrumental more than choral	kinesics (nonverbal communication)
		Time Efficiency	Productive	Higher degree	kinesics, mime, Kodaly
		Nonverbal Motivation	Attentively focused	Higher degree	kinesics, mime, Kodaly
		Positive Learning Environment	Clear and careful	Experience, females	kinesics
	Release	Group Dynamics	Interdependent	Experience, males, farther west (US)	kinesics, yoga, dance, Kodaly
		Music Concept Learning	Critical thinking	Higher levels, higher degree & experience	kinesics, yoga, Kodaly, Dalcroze
		Artistic Music Performance	Embodied expressively	N/A	kinesics, mime, dance, Tai Chi
		Student Independence	Created and felt deeply	N/A	kinesics, yoga, dance, Tai Chi
Music conducting	Control	Mechanical Precision	Timed correctly	Higher degree & years of experience	Mime, Feldenkrais, less Alexander, kinesics
		Motivational	Attentively focused	Higher degree and experience, lower levels	Mime, dance, acting, Feldenkrais, less Alexander
		Physical Technique	Produced strongly	Higher degree and experience	Mime, acting, Alexander
	Release	Expressive	Expressed musically	Higher degree and experience, farther west, males	Mime, dance, yoga
		Psychosocial	Familiarly grasped	Experience	Mime, dance, acting, Alexander
		Unrestrained Tone	Produced freely	Higher degree and experience, farther west	Kinesics, dance, acting, Dalcroze, Alexander

^†^Further informed by prior contrasts of direct vs. indirect, formal vs. informal, unsupportive vs. supportive, dependent vs. interdependent/independent, and teacher-oriented vs. student-centered (Gumm, 1992; Gumm and Essmann-Paulsen, 2001).

*The more types of methods training the more musician-oriented the teaching and conducting; no significant impact of Laban training on any teaching or conducting approaches has been detected in these studies (Gumm and Simon, 2011; Gumm, 2016a).

#### 2.2.1 Productive and correct to task

Time efficiency has the basic aim of keeping students productive or, more simply, covering more ground and getting more done. This aim is initiated by keeping instructional directions brief, direct-to-task, and focused on action, including managing multiple tasks to accomplish several things at a time. In short, it is about staying actively on task, with the amount of clock time kept on task as both the motivational incentive and criterion of success.

Assertive teaching is rooted in the earlier music education theory of sequential patterns of task presentation, student response, and feedback (Yarbrough and Price, 1981, 1989), which itself is rooted in behaviorist reinforcement theory. Assertive teaching expands and clarifies prior theory in four ways. Firstly, the pattern was refined to start with specific task directions, follow with close monitoring of response, and end motivationally with specific feedback about how students responded to directions. Secondly, the interpreted aim of the pattern is to respond *correctly* to task directions. Thirdly, motivation of correctness went beyond three sequential steps to include: communicating awareness of behavior, saying to follow directions, remind of the intended task, verbally demanding sharp attention to task, and offering consequences—though this last behavior remains in the MTSI as least definitive and reliable by its shift in focus to incorrectness. Fourthly, the added steps suggest more of an ongoing teaching cycle that sticks to the task until it is done right rather than a single sequential unit that stops at first completion.

Efficient timing and correctness are matched in conducting by mechanical precision. The conducting aim is to have the correct synchronous timing of music events. Musically, this includes beat, meter, rhythm, and the cuing of entrances at the start and cutoffs at the end of textural events. Being in time with the conductor also controls tempo, especially changes of tempo not as an expressive concern but as a matter of accuracy.

#### 2.2.2 Caring and careful learning

A positive learning environment presents a second clarifying extension to the complete sequential pattern theory that involves an even greater cyclical exchange with students. In this case, the cycle begins by clarifying student understanding of task expectations and ends motivationally with positive feedback. This form of feedback, as explained from the onset (Gumm, 1992, 1993), is called contingent praise to ensure affirmation is only given after positive learning growth is observed to occur to avoid motivationally reinforcing non-learning or negative behavior. Content from prior research on positive interpersonal relations, such as taking and answering student clarifying questions, swayed my original interpretation of its aim to foster a climate or environment for positive learning. However, the focus is on a positive-learning environment, not a positive learning-environment.

#### 2.2.3 Focused attention

Nonverbal motivation expounds on Yarbrough’s (1975) theory of magnitude or intensity of nonverbal behavior. The implied intent is to motivate attention, which explicitly is stated as a research criterion of effectiveness to get students to make eye contact and attend to tasks. Instead of the original use of high intensity alone to motivate attention, the pool of items that factored together (Gumm, 1993) highlighted appropriate shifts in facial and bodily intensity and proximity to students as the motivator. This suggests that attention can be nonverbally intensified as well as moderated, energetically enthused or calmly intrigued, uplifted or downshifted, and expanded out or narrowed in depending on observed mental focus needs at the moment.

This aim continues into a motivational conducting approach with a slight shift in nonverbal gestures. Eyes, gaze, signals, pointing, and circulating to shift proximity to musicians all aim to alert, remind, secure, and maintain attentiveness without a word. Also gleaned from observational analyses (Gumm, 2018; Gumm et al., 2018) were body leans, head turns, nods, okays, thumbs up, and other hand signals that can motivate attention in multiple directions in quick succession or at the same time. Furthermore, attention to crucial music events and their successful execution can be nonverbally alerted ahead of, maintained or reminded during, and affirmed after crucial music events.

#### 2.2.4 Embodied artistic expression

Expressive conducting is the gesturing of a particular set of concepts or features in a performance work. Music features are embodied by the conductor into expressive gestural shapes. As factored together statistically (Gumm et al., 2011; Gumm, 2016b), these include phrase contours and peaks, dynamics, accents, and other expressive score markings. It further includes reflecting the music’s overall expressive character as marked or interpreted, as well as the interpreted emotional intent of the music. Along with mechanical precision, this is a music-focused approach and includes well-timed synchronization of sound, in this case, synchronized expressive properties of the music.

Artistic music performance links to expressive conducting by both being rooted in musical score features and motivating expressivity through multisensory imagery and physical motion. It is the most unique and new approach of both models, drawing together content from research areas and theories of learning style, multisensory or cross-modality learning, modeling, metaphor, and imagery—which join together in presenting music visually, auditorily, and kinesthetically. This approach further links to physical technique and unrestrained tone conducting in helping musicians embody the ebb and flow of musical expression and further links to psychosocial conducting in the use of familiar musical imagery and metaphors.

Physical technique conducting uses motor mimicry of muscle strength, energy levels, size, and direction of musician exertions and motion to stimulate unified musical sound. Key instructional and motivational verbs in the process include directing, depicting, guiding, conveying, stimulating, modeling, and reminding of healthy musical techniques. Compared to musically focused expressive conducting, this approach connects directly with musicians to spark the physical production of sound, body to body, instead of abstract conceptual images that transfer to mind, body, and then sound, such as a raised or lowered hand to signal louder vs. quieter dynamic sound. The paired opposite to unrestrained tone, physical technique focuses on invigorating the production of heightened, peak, louder, or otherwise more intense musical sound.

#### 2.2.5 Interdependent shared influence

Music concept learning motivates critical thinking using recall and problem-solving questions. This approach draws together content from all but the highest levels of the cognitive domain along with related critical thinking research (Gumm, 1992, 1993). Learning of music concepts is shown to occur in a transactional exchange with the teacher presenting concepts and then stopping input to ask students to share their fact knowledge and knowledge-based decision-making. In the exchange, students learn from each other’s answers more than from the teacher and learn to make decisions more than follow the teacher’s decisions.

Group dynamics motivate interdependent social learning through peer leadership and cooperative strategies. The role of the teacher is to present the intended learning goal and either place individual students in the lead to present or perform for their peers or arrange small interactive groups or sectionals to work and brainstorm cooperatively. As with music concept learning, students learn from each other’s guidance, but more intentionally and inclusively, with the music teacher only involved in assuring leadership and cooperation toward assigned goals.

Psychosocial conducting uses dance, mime, acting, and other everyday gestures in the negotiation of a familiar set of gestures to which musicians will readily respond. As noted, it relates to artistic music performance in its use of everyday familiar movements. As with group dynamics, it has the intention to foster interdependent learning. As with music concept learning, it forms a transactional exchange, in this case each nonverbal gesture an open question as to how well it gets the intended response, and each musician response the answer that leads the conductor to clarify, adapt, and familiarize the gesture until the intended response is achieved. This requires vulnerability to make adjustments in musicians’ favor and be open to trying gestures not traditional to textbook conducting.

#### 2.2.6 Independent learning

In its counter role to the control of physical technique, unrestrained tone links to expressive conducting and artistic music performance teaching. The general aim is for the ensemble to make music unrestrained, yet more specifically to ease tension in physical technique, performance and social anxiety, the resulting tone color, and determination of tempo. This is done by shaping the tone in hovering, circling, flowing and lifting gestures, at most indicating tempo in nudges or shifts if not to entrust tempo to musicians. This conducting approach tinges on the full independent release of control over to an ensemble by reducing and even stopping gestures for musicians to function on their own and follow their own internal tempo. It, therefore, balances between the shared influence of group dynamics and fuller freedom of student independence teaching approaches.

Student independence involves the highest levels of the cognitive domain and components of the affective domain. These are achieved by asking for and discussing ideas and feelings about music, values and commitments to music, evaluative critiques of music, new creative solutions to musical situations, and what is important to them in music. These are motivated by encouragement and nonjudgment of feelings, creativity, and imagination. Having students share their originality is more time consuming than the caring patience of a positive learning environment. It goes beyond the correct knowledge and application of knowledge of music concept learning to the deepest levels of learning.

### 2.3 Omitted approaches and aims

Something left wholly unanalyzed is which behaviors or sets of behaviors were either left out due to weak or split loadings in initial factor analyses or replaced in subsequent validity testing (Gumm, 1992, 2004b, 2016b; Gumm and Essmann-Paulsen, 2001; Gumm et al., 2011). There are three likely reasons items did not coalesce into factors. First, they were only loosely related or off-target to the central distinguishing construct of a factor. Second, they were not part of a practice that distinguished between music teachers and conductors, which is informed by the factor analysis method I used to analyze unique variance rather than shared variance that otherwise would have identified commonly used approaches. Third is that items are loaded across multiple factors, which is due to ambiguous, unclear, or multiple intentions being understood of the same survey item by different survey participants.

#### 2.3.1 Going to extremes

Two sets of omitted items help to establish limits as to which teaching behaviors do not best fulfill the underlying learning aim of an approach. Especially they suggest how going to an extreme defeats the intended aim.

Assertive teaching stops short of demanding that students silently listen as the teacher talks, focusing on correcting errors, criticizing student mistakes, using competition to motivate learning, disciplining for inappropriate behavior, getting noticeably impatient with students, and offering rewards or punishments to get students to meet teacher demands. Omissions clarify that the focus is on asserting correctness and not being aggressive, punitive, or demeaning toward incorrectness.

On the other extreme is to stop short of being so positive and interpersonal toward students that the focus on positive learning growth is lost. This is highlighted by the omission of allowing students time to get to know each other, talk to neighbors, and help choose classroom rules, and for the teacher to admit mistakes and share personal information. Items that put the attention on student learning style differences were also omitted, including searching out individual differences, changing teaching to match the way students learn, and stopping to assist individual students. Omissions reveal the fine line between placing the focus on positive learning versus shifting the focus to the teacher being positive, students themselves, or positive interpersonal relations between the teacher and students. Certainly, a positive learning environment is motivated by students’ desire for teacher approval, praise, clarification, and accommodation. However, the caution is that it can hook students on positive teacher attention and cause them to lose focus on the ultimate aim of positive learning growth.

#### 2.3.2 Teacher talk

Of special note is how teacher talk is limited in the entire content of the music teaching model, let alone the conducting model, which is fully nonverbal. Verbal talk is limited to clear directions and corrective feedback, the briefest of directions to efficiently get to the task, clarification and affirmation, logistical directions to place individuals and peer groups in the lead, factual and critical-thinking questions to give way for student answers, musical imagery and metaphors to develop sensorial music learning and initiation of open-ended discussion for the student to express feelings and creativity. Teaching and conducting are otherwise nonverbal when monitoring student tasks and interactions, getting attention, nudging ahead using clock time, modeling by example, and nonjudgmentally observing for unique creative ideas.

This conjures a certain guiding principle to speak only as necessary to initiate and motivate an aim, then get out of the way to let learning be the primary focus. Rationally, teacher talk is a teacher/teaching action and not a learner/learning action, and it abstractly reflects back on the past or forward into the future rather than engages learner/learning actions in the present. Items in the MTSI clarify to keep teaching brief and purposefully aimed toward specific learning outcomes, thereby reducing teaching to increase learning.

#### 2.3.3 Common music skill areas

Weak loadings omitted the common music goal of physical technique from the music teaching model, including having students drill physical technique, describe performance skills, explain relationships between performance skills, and learn from the teacher’s performed examples of agility or difficult musical passages. Instead of a standalone approach and aim, omissions reveal physical technique to be a part of a coordinated pattern of music teaching aimed toward artistry—a means and not an end or smaller component used toward a deeper artistic aim.

The use of multimedia in teaching music literacy was also omitted, which would seem to relate to artistic music performance as multisensory strategies and music concept learning toward critical thinking about music. Omitted were the uses of audio and/or video recordings as students read along with the music, as musical models, and as an overhead rehearsal guide. It is plausible to view the omission of multimedia as falling outside the teacher/student exchange of music teaching or as not serving a singular, distinctive aim.

Skills of music listening and discrimination skills and the ability to think of music in their heads were also omitted. Perhaps, like physical technique, music literacy is pointed out to be a means and not a philosophical end goal in itself.

Having students probe deeper into music score analysis was also omitted. Omitted items included students analyzing the form of music, reading and translating music, determining the composer’s intentions, or interpreting the music. It also omitted content about the teacher concentrating on phrasing questions well, using probing follow-up questions, and waiting for 5 s or more after a question to allow students time to think. Sensibly, these options would belabor and distract from the central aims of making active critical-thinking choices and expressing creative and affective ideas or perhaps ambiguously fall between these aims.

Other omitted items further clarify student independence as to how far to go in developing originality. These included students comparing improvised patterns, challenging students to accept new ways of thinking, working to get students to accept and react to works of music, and helping students understand their personal feelings. Affect and creativity are shown best to be nurtured and drawn out through nonjudgmental dialogue rather than compared, challenged, worked, or helped along.

#### 2.3.4 Lesson and rehearsal structuring

Several research suggestions for organizing learning were included in the original music teaching survey. However, none made it into the music teaching model. A mix of items created ninth and tenth factors in the initial factor analysis that were not validated by confirmatory factor analysis. I called these tentative factors flexible classroom structure, with a focus on unplanned on-the-spot decisions, and sequential instruction, with step-by-step details adding up to global understanding. Omitted items included allowing students to choose when to get out of their seats and whisper and make asides.

A third set of items implying the opposite of a sequential detail-to-holistic structure was fully omitted from the music teaching model. This content included teaching abstract concepts not teachable in a detailed manner, providing an overview or key focus of the class, teaching according to a prepared set of prioritized goals, creating a climax within the rehearsal, use of whole-class learning, and having students observe and reflect. Grading by paper-and-pencil tests or performance tests also did not sort into any distinctive approaches.

Two items too weak and ambiguous to be included in interdependent group dynamics are noteworthy. Rotating rows or shuffling the permanent seating does serve to shift the dynamic interplay across a group, yet stops short of nurturing interdependence. On the other hand, motivationally appealing to an intrinsic sense of responsibility focuses on releasing control over to students yet stops short of identifying with peer-group learning.

#### 2.3.5 Separation of conducting approaches

In the development of the music teaching model (Gumm, 1993), all mention of conducting as a teaching approach was omitted. These included relying primarily on conducting gestures to communicate with students, teaching students to interpret what conducting gestures mean, and having a student conduct the group. These omissions affirm that separate research was required to more thoroughly distinguish approaches to conducting.

In developing the conducting model (Gumm et al., 2011), several music features did not find a place. Not connecting to expression as expected were heavy-to-light weighted accents, weight of tone, and articulation, the first likely due to opposite terms and all three unclear or split in purpose as either musical or physical/bodily features. In addition, resonant quality and section balance of ensemble sound failed to strongly identify with Expressive or Unrestrained Tone conducting, and right/left-hand mirroring and merging of expression within the right-hand meter pattern failed to identify with any particular aim.

Omitted from psychosocial conducting was a sense of unity with the ensemble rather than dominance, asking and drawing toward rather than requiring to happen, working to keep gestures fresh and unexpected, and dramatizing the story of the music. Split or multiple meanings can be found in each, though as well each seems to sway from the central focus of developing familiarity with gestures.

## 3 Overarching priorities

Earlier in the previous section, I concluded that different approaches are required to have an effect on different outcomes. What is more, a particular balance of approaches reveals a particular broader aim, priority, camp, school of thought, practice, or philosophy. Some add up and balance toward historical, philosophical aims, others merely show how certain approaches play a subservient role to the aim of a top-ranked approach, yet others have unique underlying aims that I did not find to match common priorities within music education.

### 3.1 Control and release

#### 3.1.1 A new dichotomy out of old

Complementing previous theories and philosophies, higher-order factor analysis of music teaching approaches revealed and validated a unique pair of overarching aims that joined teacher- vs. student-oriented, extrinsic vs. intrinsic, and active vs. reflective dichotomies into dichotomous aims to cover breadth versus uncover depth (Gumm and Essmann-Paulsen, 2001; Gumm, 2004b). Later, in factoring both models’ approaches together, the same pairings of teaching approaches aligned with conducting approaches in a way that suggested a new duality—to control or release (Gumm, 2016b; see Tables 1, 2).

Control is achieved in teaching by verbally asserting correct responses to task directions, nonverbally motivating attention to task, being quick and efficient to task, and affirmingly reinforcing positive learning growth. Control is achieved in conducting by being time precise, gesturally motivating attention, and guiding physical music-making efforts.

Release is achieved in teaching by an interdependent peer-learning group dynamic, asking conceptual questions, connecting artistic sound and physical motion, and nurturing independent ideas and feelings with nonjudgment. In conducting, the release is the common focus behind expressive, psychosocially negotiated, and unrestrained or tension-freeing gestures.

To be clear, there is no domineering or demeaning connotation in my choice of terms. Control is more about narrowing or drawing attention inward and toward, chiefly by extrinsic teacher/conductor control. In contrast, release is about broadening attention outward or letting go toward intrinsic outcomes.

#### 3.1.2 Average priorities

Of these two overarching priorities, control is consistently shown to be most prevalent on average. Upfront, this seems due to both models originating within a context of ensemble teaching and conducting that mostly involves active learning aimed toward performance (Gumm, 1993, 2016a,b; Gumm et al., 2011).

Control remains most prevalent on average across all geographic regions studied. This includes research in the U.S. southeast (Groulx, 2010), mid-Atlantic (Gumm, 2004a; Basilicato, 2010; Anderson, 2013), north-central (Brakel, 1997; Gumm, 2004b, 2007; Bazan, 2011), southwest (Gumm and Essmann-Paulsen, 2001), and across mixed regions (Gumm, 1993, 2016a,b; Olesen, 2010). It also includes research outside the U.S. in Malaysia (Shah, 2005, 2007), Taiwanese in Canada (Hsieh, 2010), and Japan (Courtney, 2014).

Control of active behavioral-task learning has also remained the stable and persistent focus across generations. This observation is based on national studies of choral directions 25 years apart (Gumm, 1993, 2016a) and even when expanded to include conductors of all types of ensembles in multiple U.S. regions (Gumm, 2016b). Across the quarter century, however, differences were less polarized, reflecting overall less assertiveness and increased positive learning on average.

Control bears out to be the top priority in elementary music as evidenced in one study (Shah, 2005, 2007). Yet, release approaches are shown to be of higher priority in elementary than secondary music as evidenced in another (Bazan, 2011). The latter is a relative and not a top-priority finding; nonetheless, these expose a dearth of research applications of the MTSI and CPS in general music, whether elementary or secondary, and none in ethnic, folk, popular, community, or professional ensembles.

### 3.2 Diverse philosophies

In the variance on both sides of the mean average, a hidden diversity of philosophical practices was revealed (Gumm, 1993, 2016a). Applying cluster analysis, MTSI scores were sorted by commonalities among and between directors to reveal smaller groups who coordinate and balance teaching approaches toward a shared practice, common overarching aim, or educational philosophy.

#### 3.2.1 Music performance

Five cluster groups reveal different degrees to which music performance is prioritized. Each balanced different types and levels of control with and without the release of deeper learning.

The first two reveal a slight shift in focus in managing on-task behavior. The first group I called Task Oriented (Gumm, 1993) by moderate priority toward control of active task learning topped by efficiency and correctness. With no high-priority learning aims, it seemed a basic aim to be correctly on task more of the time. Of note, 25 years later, a group I called Task Nurturing (Gumm, 2016a) retained efficient productivity as the top priority yet showed less assertiveness and more nurturing of positive learning—reflecting the same shift as revealed on average between the 1990 and 2015 samples.

One group in the 1990 sample enhanced a traditional music performance approach with a top priority toward embodied artistry, underlaid by nonverbal attention-getting and corrective approaches. The lowest priority toward time efficiency seems appropriate given how artistry requires a more start-and-stop use of time to link sound and body toward expressive musical performance. I interpreted their global aim as most fully Music-Performance Oriented. No group in the 2015 sample showed a similar balance or global aim.

Priorities toward even deeper learning aims centered around music performance were found 25 years apart. The earlier group I called Discovery Oriented for its top mix of priorities toward group dynamics and artistic music performance, followed moderately by student independence with support of nonverbal motivation (Gumm, 1993). Learning seems focused on self-discovery through shared contributions, the embodiment of expression, and feelingful and thoughtful responses guided silently with teacher enthusiasm. In the 2015 sample (Gumm, 2016a), a group cluster of choral directors I called Engaged Discovery enhanced the artistic embodiment of music and independence of feelings and ideas with an engaging question-and-answer exchange and more efficient use of time in place of shared group leadership.

#### 3.2.2 Comprehensive musicianship

Four groups showed even closer coordination between conceptual, artistic, and independent learning—the pillars of perform, analyze, and create that define comprehensive musicianship. The key difference between these groups shows a merging of two prior dualities: performance vs. comprehensive musicianship and teacher- vs. student-oriented.

Within the 1990 sample (Gumm, 1993), the assertion of correct behavior was given low priority by the first comprehensive musicianship group and high priority by the second. I called these Student-Centered Comprehensive Musicianship Oriented and Teacher-Controlled Comprehensive Musicianship Oriented.

Then, in the quarter-century follow-up study (Gumm, 2016a), the polarity of teacher vs. student dissipated into what I called Shared-Influence Comprehensive Musicianship and Energetic Comprehensive Learning. The first had a complementary balance of positive, efficient, and motivating approaches, with assertiveness well in balance as well. The second supported comprehensive priorities by being highly positive and nonverbally enthused yet far less assertive. The decreased polarity coincided with less focus on asserting correctness and a greater focus on positive learning and time efficiency across the national sample.

#### 3.2.3 Conceptual learning

To teach about music being performed was a key philosophical counter aim to traditional performance dating back decades in music education (Gumm, 1992). This was found to be a priority in three cluster groups.

Surfacing in the 1990 sample (Gumm, 1993) were a Concept- Presentation Oriented group and Content Oriented group. The former was similar to Task Oriented in its moderate priorities toward asserting correct and efficient task learning, yet was supported by conceptual questions. This met the philosophical aim of teaching about music being performed in its simplest form. The latter group delved into the content of music with frequent use of all three inquiry approaches aimed toward clear and positive, music concept, and independent affective/creative learning. Less frequent use of assertiveness, peer-group learning, and efficiency served to nudge active and interactive learning in the balance.

A group in the 2015 sample (Gumm, 2016a) that I called Active Concept Application supported music concept learning with frequent use of positive, nonverbal, and efficient approaches, and moderately linked conceptual learning with artistic and affective/creative aims. This balance seemed strongest in fulfilling the philosophical argument to learn to perform with deeper meaning and understanding under the guidance of a music director.

#### 3.2.4 Cooperative learning

Group dynamics took the forefront in clusters detected in both studies. One 1990 group of directors relied on group dynamics almost to the exclusion of teacher involvement except to keep students efficiently active and ask conceptual questions, both at lower levels by comparison (Gumm, 1993). This group I called Student/Subject Matter Interaction Oriented for simply letting students learn from each other to actively do and reflectively think about music.

Another group prioritized positive and independent learning, peaked by cooperative peer-group learning. These slower-paced approaches were tempered with moderate efficiency, assertiveness, and silent visual motivation by the teacher and enhanced by less frequent conceptual questions and artistic strategies in a rich balance fit to a Cooperative Learning Oriented philosophy.

Even more pointedly balanced toward group dynamics was a 2015 group focused on Cooperative Affective Learning (Gumm, 2016a). A secondary mix of performance artistry and independent thinking and feeling have a commonality of musical affect, the former embodying emotionally expressive music performance and the latter facilitating the verbal expression of feelings about music.

#### 3.2.5 Less teacher

Earlier, I pointed out how limited mention of teacher talk implies that putting less focus on the teacher teaching keeps the focus more on learners learning. Two final cluster groups, both in the 1990 sample (Gumm, 1993), inform how this works out and how it does not.

First, a group I called Low Teacher Involvement Oriented had the commonality of having students share their affective, creative, and conceptual grasp of music in clear, affirming, and cooperative interpersonal exchanges. These music directors remained uninvolved in controlling learning assertively, nonverbally, or efficiently in favor of deep intrinsic learning. These teachers, then, taught less so that learners may learn more deeply.

Second was a group I interpreted as Nonfocused Low-Interaction Oriented for being flipped upside down from the other. This group was uninvolved toward interdependent, independent, conceptual, and artistic learning aims, with ratings for positive, corrective, efficient, and nonverbally motivated learning as well so low as to suggest they were intentionally aloof and unfocused toward any particular learning aim much at all.

### 3.3 Sources of stability

I return to the issue of stability again in the context of stable patterns of approaches. The discussion here is how long and in what contexts do unique philosophical priorities linger.

#### 3.3.1 Underlying latent traits

Three studies support the concurrent validity of the MTSI and CPS in measuring stable and pervasive philosophical priorities. Both rating-scale surveys were verified to measure stable latent traits underlying in-the-moment choices across time.

Though seeking to qualitatively explore band directors who scored as being student-oriented on the MTSI, as noted earlier, Bazan (2011) instead found a pervasive teacher-directed focus across the sample. Observations validated the greater priority toward teacher control, yet not much in the way of releasing deeper student learning. Interviews, however, revealed how they used freer, releasing, deep-learning approaches in early rehearsals—including after concerts early on in the next concert cycle. The greater control came closer to a concert—which was when Bazan observed them. Viewed long-range, the MTSI validly captured the overall balance of priorities weighed more toward control than the short-range focus on release. Their reported priorities panned out as being stable across the stretch of multiple concert seasons.

Second, a seasoned band conductor’s (Gumm, 2018) and choral director’s (Gumm et al., 2018) self-perceived conducting priorities on the CPS correlated strongly with the balance of gesture functions observed across a series of randomly chosen rehearsal episodes. Results validate the ability of the CPS to predict a long-range balance of priorities, in both cases, across the span of one or two rehearsals.

#### 3.3.2 Place and culture

Research has further shown how the stability of teaching and conducting approaches is anchored in geographic location and cultural traditions. In short, seemingly unique and changing choices are stably grounded by the accepted practices of those around us.

There is an east-to-west drift in priorities across the U.S. corroborated by several studies. Positive interactions toward clear and careful learning take top priority along almost all of the east coast, with time efficiency falling and assertion of correctness rising in rank from southeastern to northeastern states (Gumm, 2004a; Basilicato, 2010; Groulx, 2010; Anderson, 2013). Interdependent cooperation and independent learning were the lowest priority in mid-Atlantic states (Basilicato, 2010). Gumm (2003b) found that choral directors farther east were significantly more assertive and nonverbally motivating yet nurturing of positive learning and artistry. In contrast, directors farther west were more supportive of peer-group dynamics and student independence. However, only peer-group learning continued to be a greater Western priority 25 years later (Gumm, 2016a), yet an east-to-west trend toward greater unrestrained and psychosocial ensemble autonomy in conducting adds to a general drift toward supportive instruction farther west.

In the upper central region of the U.S., Brakel (1997) found select band directors to be the most time efficient regardless of individual differences of gender, degree level, or school size. Bazan (2011) found band directors in the region to place the most emphasis on time efficiency, with other controlling approaches following in rank. Choirs in the region, however, ranked their directors as being most positive and efficient, followed by nonverbal motivational and assertive control (Gumm, 2004b). University ensemble members associated efficient and positive approaches with professors viewed as most helpful to their learning and independent learning as preferable over assertively correcting them (Gumm, 2007). This same nudge toward efficiency was found in select Kansas school bands (Gumm and Essmann-Paulsen, 2001), followed by assertive control and then affirmation of positive learning in rank order.

This tradeoff between positivity and efficiency takes on cultural significance in international comparisons. Hsieh (2010) profiled Taiwanese private music teachers in Canada, finding a contrast between conceptual, artistic, and positive approaches suited to Western cultural values and an efficient and assertive approach focused on obedience suited to traditional Chinese/Taiwanese cultural values. Japanese band directors were shown to emphasize assertive teaching, nonverbal motivation, and group dynamics more than a comparative sample of U.S. band directors who put more emphasis on a positive learning environment, music concept learning, and student independence (Courtney, 2014). Time efficiency was a similarly high priority for both.

#### 3.3.3 Context

Finer contextual situations have been found to further anchor instructional and motivational priorities in place (see Table 2). Most circumstances of career positions are shown to steer instructional and philosophical priorities in distinct directions.

The first contextual anchor is the size of the program. Brakel (1997) found significant correlations between band size and positive learning (inverse) and artistry learning aims, and a music-concept approach and school size, perhaps the lower general affirmation a reflection of more intentional deeper music-learning goals with larger groups. On the contrary, in the smaller geographic region, Bazan (2011) found that the larger the band program, the more assertively and efficiently band directors taught, and the smaller the school enrollment, the more nurturing directors were of independent feelings and ideas. With directors of mixed ensemble types across mixed regions, Gumm (2003b) identified school size as being of significant influence on assertive and nonverbal motivational control and peer-group, concept learning, and student independence release, which altogether suggest larger schools provide for greater exploration of varied learning aims.

Second, rehearsal time and timing are implicated as having an influence on priorities. Bazan (2011) found greater affirmation of positive learning in directors who have fewer numbers of rehearsals and that directors were more nonverbally motivating and inquisitive in asking conceptual questions in ensembles with a greater number of concerts per year. As previously noted, Bazan also found priorities to shift from releasing to controlling approaches in rehearsals farther to closer away from concerts. Furthermore, directors with a greater number of performances were found to affirm positive learning and motivate nonverbally more.

Third is the grade or experience level taught. Gumm (2016a) found that assertion of correctness and nonverbal motivation of attention both in teaching and conducting lowered across choir levels. This reveals that as students advance, these burdens on teachers decrease. With directors of a range of ensemble types, Gumm (2016b) found that those who most conduct ensembles higher in grade, age, or experience level seem to sway toward expression. This also shows greater release as students advance in grade level. Research has yet to study adult community and professional ensembles of varied levels of ability and experience.

Fourth is the type of ensemble most centrally taught. Findings in relation to conducting (Gumm, 2016b) suggested that a career position focused on teaching instrumental, choral, or general music seems to anchor how much or little control is taken over musical precision and a musician’s physical technique. Conductors at the university level demonstrated distinct priorities logical to the nature of the ensemble, especially higher priority toward precision in orchestra and physical technique in band and choir (Gumm, 2018; Gumm et al., 2018). Sorting MTSI research by ensemble type, bands seem most efficiently focused (Brakel, 1997; Gumm and Essmann-Paulsen, 2001; Bazan, 2011), even more so with Japanese bands (Courtney, 2014), and choirs consistently place positive learning above efficiency (Gumm, 2003b, 2004b, 2016a). This is yet another issue that requires further research to sort out. However, on the surface, such results make sense to the general historical band and choir traditions. Further research is also needed to see how traditions of other types of ensembles pan out in practice, such as contemporary pop a cappella as well as folk and ethnic music ensembles.

### 3.4 Sources of change

It is in the relative portions of control approaches and precise mix of release approaches where developmental changes in music teaching and conducting are found. What seems to shift priorities is our experience, situation, and background (see Table 2).

#### 3.4.1 Career experience

As noted earlier, priorities toward control remained relatively stable over the years in the choral music field, yet relaxed slightly by way of reduced assertiveness and less polarized differences between philosophical aims. However, a clear and consistent pattern is evident when it comes to individual change across years of experience—a pattern that I can plausibly describe as deeper, freer, and wiser (see Table 3; Gumm, 2003b, 2004a, 2016a; Bazan, 2011; Gumm and Simon, 2011).

**Table 3:** Trends of career experience development.

	Teacher dependent		Interdependent	Student independent
	Years 1–2 Self-Reflective “Novice” Stage	Years 3–7 Broadening “Competent” Stage	Years 8–9 Shared-Influence “Letting Go” Stage	Years 10–19 & 20+ Deepening “Wise Choices” Stages
Advantages	Beginner’s enthusiasm & eagerness Creativity and spontaneity	Time efficiency--less wordy, quicker to task Assertive teaching--more externally aware and in control of classroom behavior, able to hold students to task Motivational conducting—attuned to attentional needs, opens awareness toward next stages of development	Group dynamics—shares the burden of decision making by developing student shared leadership Psychosocial conducting—vulnerably adapts so gestures are familiar	Generally more releasing as with earning a higher degree A new aim for students to learn to learn on their own, more deeply and meaningfully Time efficiency and gesture precision become more pertinent and coordinated to varied learning aims On balance, more expressive with an integration of physical technique and unrestrained conducting
Problems	Inefficient time management Wordiness—slow to task Potential burnout with growing efficiency and assertiveness Less expressive in conducting	Quick paced controlling approach can be task-oriented and lack deeper learning aims The tightly controlled environment puts a heavy decision-making burden on the music teacher/conductor Together these may lead to increased burnout symptoms		Student self-responsibility and shared leadership, taken to an extreme, may lead contributing less, being distant and aloof, and less connection with students

In brief, music teachers start out inefficient yet rather experimental in trying out different approaches; in the first 3 years grow more efficient; by year eight come to share control by facilitating peer-group leadership; by 10 years and increasingly starting year 20 grow significantly more conceptual, artistic, interdependent-group, positive, and efficient. As shown in Table 3, I call these a novice *Self-Reflective Stage* for the greater focus on self in the new teaching role, a competent teacher-centered *Broadening Stage*, a letting-go *Interdependent Transition*, and seasoned or veteran mid-to-later-career *Deepening Stages* more fully freeing of student learning.

Time rises into consideration again in the context of career development. Gumm (2003b, 2004b) found that choral directors of less experience prioritized time efficiency most—as they first worked to gain control. Experienced teachers then grew less time driven.

Gumm’s (2016a) findings 25 years later support this pattern of development. With increasing years of experience, directors grew more nurturing of positive learning and more releasing in the use of interactive group dynamics and conceptual questions. Foremost, nonverbal motivation—though a method of control—was revealed to serve as something of a gateway to greater release of control, that perhaps sharper awareness of students’ and musicians’ attentional focus opens awareness to their deeper self-regulated capabilities.

However, other researchers corroborate only certain facets of career growth. Brakel (1997) and Olesen (2010) corroborated only an experience-based shift toward peer-group or peer-led learning. Bazan (2011) found a strong correlation between the use of peer-group leadership and years of experience and years in the present position and a negative experience-based correlation with positive learning. Anderson (2013) found no differences in music teaching approaches based on years of experience, though the fact that “Equal representation with respect to directors’… years-of-teaching experience were limited” (p. 79) casts doubt on this contrary finding. Again, despite the more substantial concurring evidence, further research is required to assure these facts and interpretations are generalizable.

Experience-related shifts in conducting have been studied less, leaving future research to sort out more thorough patterns of development. Gumm and Simon (2011) found that motivational and physical technique conducting approaches increased with years of experience. Gumm (2016b) corroborated expressive and unrestrained conducting as the two key developments across years of experience. Though I sorted current findings logically in line with music teaching stages (Table 3), future research of the conducting model is required to empirically sort out progressive stages of development.

#### 3.4.2 Impactful experience

Education is a form of experience that is shown to be quicker in changing well-anchored pedagogical approaches and philosophical aims. Research has uncovered movement training, educational degree earned, and workshop training as influencers of change (see Table 2).

First, movement methods were shown to have a modest but significant and widespread impact on conducting priorities, with training in multiple methods shown as a quicker route toward being a more musician aware and responsive conductor. Gumm and Simon (2011) revealed and Gumm (2016a) corroborated how responsive musician-oriented conducting develops through combinations of movement methods. Effective in swaying priorities in directors of a range of ensemble types (Gumm, 2016a) were dance in relation to expressive, motivational, unrestrained tone, and psychosocial conducting priorities; Alexander technique toward mechanical precision, physical technique, unrestrained tone, and psychosocial conducting priorities; acting with impact on physical technique, unrestrained tone, and psychosocial conducting; Feldenkrais toward precise and motivational conducting; and Dalcroze Eurythmics on unrestrained conducting. With choral directors alone (Gumm, 2016a), precise conducting was lower for Alexander and higher with mime, and expressive conducting was higher with yoga and mime; motivational conducting was lower with Alexander and higher with mime and acting, psychosocial conducting was higher with mime, physical technique conducting was higher with mime and acting, and unrestrained conducting was higher with dance, kinesics, and Dalcroze Eurythmics. The general movement method of Laban was not shown to sway priorities in conducting.

Toward shifting choral directors’ teaching priorities (Gumm, 2016a), mime impacted nonverbal motivation and artistry; Kodály impacted nonverbal motivational, efficient, peer group, and conceptual aims; kinesics influenced all but assertive teaching aims; Dalcroze influenced higher priority toward conceptual learning; yoga impacted peer group, conceptual, and independent learning aims; dance had an effect on peer group, artistry, and independent aims, and Tai Chi impacted artistry and independent learning aims. Teaching priorities were not found to significantly vary by music pedagogies of Gordon or Orff, the general movement methods of Alexander, Feldenkrais, or Laban, nor by training in acting. What this suggests is that while certain name-recognized pedagogies may be adopted for their particular benefits, they are not shown to shift or sway choral directors’ overall teaching approach one way or the other. Olesen (2010) similarly found that abiding by the aims of an existing method—in this study, a particular warmup method—showed little relationship with the music teaching approach as measured on the MTSI except perhaps for warmups to guide independent thinking across the subsequent rehearsal.

Second is the quicker impact of an advanced degree relative to and separate from years of experience. Brakel (1997) found select band directors in a region of a single state to not have developed different priorities based on degree level. In the 1990 national choral directors sample (Gumm, 2003b), degree level likewise was not found to predict higher priority toward music teaching approaches, though it was associated with lower festival ratings. Higher degree levels in the 2015 choral director sample predicted greater nonverbal motivation, time efficiency, and music concept learning in teaching and higher priority toward all but the psychosocial factors of conducting (Gumm, 2016a). With directors of varied ensembles (Gumm, 2003b), higher degree levels showed heightened priorities toward expressive and unrestrained-tone conducting approaches.

Third, professional development clinics and workshops are an alternative that has a relatively small impact, as shown in the initial 1990 sample of choral directors (Gumm, 2003b). In the study, workshops were found to have a slight effect on increasing artistic music performance, as well as being linked to directors who participated in festivals.

### 3.5 Different perspectives

Research of these music teaching and conducting models reveals how perceptions of the actions of the same teacher or director differ when viewed from different perspectives. In this section, we compare the external perspectives of the ensemble and expert observer with the internal self-perceptions of music teachers/directors, as well as that of resulting outcomes.

#### 3.5.1 Music teacher/conductor

Music conductors have been found to not consciously grasp all of their own actions right up front. In video-recall interviews (Gumm, 2018; Gumm et al., 2018), conductors initially noticed and described only those approaches that met conscious priorities, then in subsequent viewings of themselves, added prompts to look for gestures fit to each of the model’s six conducting aims sparked recognition and affirmation of previously unnoticed gestures—including ones conductors had previously described as philosophically opposed to their conscious efforts. This suggests that much of the rapid decision-making in conducting is reflexive and subliminal, taking no time for conscious thought to react in the moment.

Even so, conductor self-descriptions came to highly correlate with CPS self-ratings after summing unprompted conscious self-observations with prompted realizations of self-behavior. This suggests that in the process of rating gestures item by item without foreknowledge of which fits in a pattern with others, the CPS validly sums up conscious and unconscious priorities. This fits the nature of latent—which means hidden—traits that underlie human actions.

#### 3.5.2 Students/musicians

Two avenues of research inform student ensemble musicians’ external view looking back at the educator/conductor. First is student actions or decisions in response to conductors with different priorities, and second is asking musicians to rate and describe their conductors.

Student retention and attrition are informative indicators of student responses to a conductor. Brakel (1997) found that no single music teaching approach predicted attrition and retention in instrumental programs but that combinations of approaches did have a significant predictive effect. Specifically, traditional directors with aims to control behavior had higher dropout rates, and directors who developed independent autonomy and performance artistry to greater extents had higher retention. The fact that coordinated approaches predicted students staying or leaving supports the greater motivational role of the implicit priorities and philosophy in the pattern of teaching.

Ensemble musician MTSI ratings of their conductors were found to differ by how they uniquely learn and are motivated. Ensemble members in two bands (Gumm and Essmann-Paulsen, 2001) and four choirs (Gumm, 2004b) were found to rate directors as foremost controlling of active learning and rate themselves as motivated by personal effort and musical ability to succeed in music. Directors’ efforts to keep musicians actively engaged in music are precisely what would catch the attention of students able and willing to put active effort into succeeding. Yet differences in perceptions of directors surfaced when motivational attributes were broken down. Those who noticed controlling approaches to greater extents were musicians motivated more by effort, musical ability, class environment, and preference for music over other activities and less by personal commitment. Musicians motivated by their affective enjoyment of music noticed positive, group dynamic, and conceptual learning aims to a greater extent—all of which allow students to share what they enjoy about music. Those motivated by their experiential and family background in music also noticed peer-learning group dynamics to greater extents, as well as strategies aimed toward artistic music performance and independent learning—all of which let them apply their unique background experiences. In addition to noticing controlling approaches, those motivated by the ensemble class environment to greater extents also noticed directors’ efforts to promote interactive class discussion of independent feelings and ideas to greater extents.

Comparing student MTSI and university faculty evaluation survey ratings, Gumm (2007) found band, jazz band, orchestra, and choir students evaluated efficient and positive teaching approaches as most effective and helpful toward their learning and getting to share their independent insights as more preferable over assertively being told what to do. Artistic performance strategies were viewed as being better prepared and being asked to interactively learn from their peers as being less prepared yet a trait of a more accessible conductor. Comparing self-attributed motivations to succeed in music, more experienced, able, and effort-oriented musicians thought less of conductors’ positive interpersonal sociability. In contrast, those motivated by affective enjoyment of music thought more of conductors’ sociable respect.

Research on conducting priorities (Gumm, 2018; Gumm et al., 2018) has found student views of conductors to be reliable, valid, and consistent with either the conductor or expert observer, seemingly due to either the career stage of the conductor or ensemble type. Ensemble CPS ratings correlated (a) strongly with observation analysis and CPS ratings of an early-career conductor, (b) very weakly with observations and moderately with CPS ratings of a late-career conductor, and (c) strongly with researcher observations, moderately strongly with conductor interview content, and strongly with a random member’s interview content. In interviews, the early-career choir director and singers grew more conscious of gesture intent with increasing stages of prompts. In contrast, the late-career choir and band conductors and musicians were more directly conscious of similar functional gesture intentions, which the choir conductor explained were developed and pre-planned.

#### 3.5.3 Expert observer

Earlier, it was pointed out how limited observations did not representatively capture the balance of approaches, aims, and priorities across broader swaths of time (Bazan, 2011). Also pointed out earlier was how representative observational procedures correlated differently with measures from other points of view depending on conductor experience level and perhaps ensemble type (Gumm, 2018; Gumm et al., 2018). An expert observer’s view has been shown to correlate highly with ensemble surveys of an early-career choir director and late-career university band director—pointing out their similarity as external views looking in on the conductor—or correlate weakly with ensemble ratings of a late-career choir director.

On the other hand, an expert observer can sum up priorities similar to self-ratings and self-descriptions of experienced band and choir conductors—pointing out their similarity of expertise—yet weakly with self-ratings and interview content of a choir director in the early years of university conducting. Though relatively scant to this point, this evidence corroborates experience-based findings across the body of research.

#### 3.5.4 Effects and outcomes

Effective approaches to teaching and conducting typically are linked to a select criterion of effectiveness or success. Neither the music teaching nor conductor models were developed in relation to preselected criterion measures, and instead, the criterion or aim was interpreted as factored patterns. Nonetheless, certain outcomes have been found when higher priority is shown toward select approaches.

Measures of student learning would serve as a direct indicator of effective music instruction, an option only loosely applied to the music teaching model. Bazan (2011) found that the students of band directors who facilitated peer-led learning more frequently had significantly lower standardized test scores; this approach was more likely an accommodating remedial choice rather than a cause of lower test scores. Anderson (2013) found that efficient, independent, and artistic (inverse) learning approaches significantly correlated with students’ self-reported learning success. This suggests that a balance of staying efficiently on task and having students explore their unique feelings and ideas either promotes student learning overall or attracts higher achieving students and perhaps artistry attracts or assists less successful learners.

A cautionary finding is a career outcome found to occur with over-emphasis on assertive and efficient teaching—increased symptoms of teacher burnout (Gumm and McLain, 2013). Both approaches together require quick and numerous decisions to the point of potential burnout. This pairs with the detrimental effects of control-oriented teaching on student dropout (Brakel, 1997). Music teachers with fewer burnout symptoms may have been less controlling in the first place or, as implicated by experiential trends, may have overcome burnout and survived in the profession by learning to be more releasing.

A traditional measure of effectiveness is the rating received at festivals and competitions. Initially used to establish the predictive validity of the MTSI (Gumm, 2003b), higher choir festival ratings were achieved by choral directors who applied nonverbal motivational and artistic music performance strategies to greater extents. In contrast, Olesen (2010) found that it was a balance between time efficiency and student independence, with less assertiveness, that predicted choral directors’ professional and choir success. In further contrast, band directors who earned higher competitive band ratings were found to be more efficient and conceptual, more assertive, and less nonverbally motivating (Groulx, 2010). Directors who had their band compete more were also more efficient and conceptual, yet less correctively assertive, positively reinforcing, nonverbally motivating, and group interactive. Contrasts require further research to resolve whether certain findings are spurious or due to contextual differences between ensemble types, regions, or time constraints.

A less subjective performance outcome to expert ratings at festivals and competitions is the objective ensemble sound properties that result from different combinations of conducting approaches. This has been analyzed of only one conductor in one available study (Gumm, 2018), in which noise and harmonic dissonance, tone color, volume, and loudness levels were shown to fluctuate in logical relation to observed conducting approaches. For example, motivational gestures shifted tone and volume, physical technique gestures increased sound and broadened and sharpened tone, and unrestrained gestures reduced noisiness and loudness and darked and refined tone.

## 4 Discussion

The purpose of this study was to summarize the research of Gumm’s empirical models of eight music teaching and six conducting approaches and their application in the profession across three decades toward varied philosophical aims. Synthesis of distinct results led to a hierarchical structure across this writing, revealing how behaviors coordinate into approaches, approaches coordinate into overarching priorities, and overarching priorities link to philosophical practices and schools of thought. Discussion of insights, conclusions, and future research directions—in addition to those posed earlier—follow this same structure.

### 4.1 Approaches

Key insights from this body of research are that each distinct approach motivates a unique learning outcome, unique learning aims are motivated in unique ways, and therefore, motivation of music learning depends on the intended learning aim. Furthermore, different approaches are required to have an effect on different outcomes. The conclusive lesson is to not merely apply a conducting gesture or teaching behavior situationally but tied to a broader mindful aim in coordination with other relevant gestures and behaviors.

To be clear, I would not conclude to say that content items omitted in initial model development are not done or not to be done, just that practicing music teachers and conductors, on the whole, did not report using or coordinating these behaviors consistently, strongly, and distinctively enough to count. This is a key limitation in this body of research, that all results are based on self-perceived behavior—yet corroborated from other perceptual viewpoints—and on the assumptions designed into factor analysis and other descriptive and multivariate statistics. Omissions help distinguish the diverse professional practices of a diverse range of practicing professionals, nothing more.

### 4.2 Overarching priorities

Particular schools of thought or philosophical aims are put into practice through a particular combination of behaviors toward a particular combination of approaches. If for nothing else, this body of research shows how historical philosophies have been implemented and practiced in real time and real contexts. Of historically recognized single-aim dualistic approaches, most were refined and elaborated more than confirmed outright, helping to clarify a delicate balance in their meaning and intention. However, research has yet to identify cluster groups of conductors by similar conducting philosophies, the same as done with the music teaching model, chiefly because the model accounted for pre-existing trends identified in the field of research.

Geographic findings seem to align with historical, philosophical trends, with eastern and north central U.S. being where the strict performance ensemble tradition in the U.S. first originated and spread. Western U.S., more at a distance from performance ensemble roots, shows a loosening of control toward more creative and effective intentions, something like a stereotype of its historical character as well. Once established, a tradition is conserved by continuing educational practices, as the evidence seems to support. However, continued research is still warranted in tracing shifts across time, place, culture, and other individual differences. If for no other reason, this research helps toward understanding the roots of our identities, sense of purpose, and place in our profession, whether following along or striking out in seemingly new directions.

It is plausible that deeper control-releasing approaches do not add up as higher priorities in early-career music teachers because related behaviors are not used in coordinated ways. It is only when the full complement of behaviors is rated highly that it would rise to a higher rank in the balance. Therefore, the difference may be that experience leads to more coordinated and focused teaching around deeper teaching and musician-oriented teaching and conducting. A plausible alternate interpretation is that seasoned teachers come to see behind the extrinsic exterior of behavior to see intrinsic or internal subtleties underlying student behavior. Development of releasing approaches may also take time and experience because they are more subtle and not consciously comprehensible to preservice and early-career music educators—whose primary need may simply be to learn how to extrinsically take control.

A concern posed by this body of research is whether the active, breadth-oriented, controlling approach consistently found most common on average reflects a relatively low proportion of music teachers who persist in the field—that control as an early-career trait skews the average. The average accepted approach across the profession may simply reflect greater numbers of novice and early-career music teachers and fewer of greater experience. This further suggests that plans to develop deeper learning across grade levels may be stymied by a lack of experience to carry them out. Or perhaps such deeper, long-range learning goals are put into place by professionals of longer experience. It may simply take a long experience to manage deepening goals across students’ long-range school experience. Music teacher retention is, therefore, highlighted as a crucial concern in meeting national core standards and other differentiated and deep-learning educational goals.

### 4.3 Pedagogical implications

In this balancing of approaches, a conclusive lesson is that there is so much more to music education than getting music students and ensemble musicians to follow the music teacher’s or director’s lead. All presumptions that music teachers and conductors are dictatorial and authoritarian are quashed by this research. Our profession needs to learn from our very finest, who come to learn from learners. We all can tap into our learners’ unique intrinsic feelings, ideas, influences, and abilities at any time. On balance, just as there is musical fall and rise, contraction and expansion, downbeat and upbeat, loud and quiet, and harmonic and cadential tension and release, so too can music instruction restrain and unrestrain, downshift and uplift, narrow in and widen out, grab hold and let go, and intensify and ease off. All that is needed is to ask, discuss, group up, reduce or stop moving, and check in to find what is being felt, understood, and able to be done collectively or alone—which then better informs and customizes what and how to teach or conduct.

This leads to a suggested sequence across educational and professional development programs. First are the specific instructional and motivational behaviors of each approach (see Table 1) for music teachers and directors to learn to coordinate toward purposeful aims (see Table 2). An evidence-based sequence (see Table 3) would be first to develop how and when to focus learning behavior toward productive, correct, attentive, and clear and positive learning aims. Scanning a music class or ensemble for in-the-moment needs and accomplishments and responding with nonverbal alerts, nudges, and affirmations opens the gate toward forming deeper bonds, connections, unity, and flow states, all linked as one. The transition to deeper stages then starts by simply drawing out exemplary musicians to help set the example, adapting musician motions and ideas into conducting and teaching decisions, and asking peers to help influence and be influenced by each other’s understandings and performance movements. This would be followed by approaches aimed toward artistically expressive, interdependent, or independent learning aims. Such a sequence could be applied by professional development planners in providing workshops suited to varied developmental needs and experience levels and by school administrators in evaluating effectiveness in relation to appropriate developmental trends. Overarchingly, career development seems a matter of learning to coordinate behaviors toward the specific aim of each approach and to coordinate approaches toward a broader philosophy.

A subsequent implication is a deeper insight into the powerful instructional and motivational roles of a teacher or conductor. The key to motivation is to draw attention to specific and intentional forms of learning and not place attention on non-learning such that it may motivate more non-learning by heightened attention and highlighted examples. In short, the implication is that whatever is motivated to attention grows.

These multivariate models of music teaching and conducting are shown to be serviceable, functional, matter-of-fact, purposeful, thorough, and practicable ways to put philosophy into action. Knowingly or not, whatever is motivated to attention also motivates a particular philosophy of music education. Toward making decisions more conscious, the eight music teaching and six conducting approaches are empirically validated as pedagogical foundations ready to implement the historical, philosophical, and psychological foundations of music education.

## Author contributions

AG: Writing – original draft.
